# Incidence and Outcomes of Endotracheal Intubation in Patients Presenting With Acute Poisoning to the Emergency Department of a Tertiary Care Center in India: A Prospective Observational Study

**DOI:** 10.7759/cureus.117325

**Published:** 2026-10-01

**Authors:** Sreevardhan Reddy Ambati, Poonam Arora, Bharat B Bhardwaj, Parampreet Singh, Sarath S Nair, Koushik D

**Affiliations:** 1 Emergency Medicine, All India Institute of Medical Sciences Rishikesh, Rishikesh, IND

**Keywords:** acute poisoning, emergency department, endotracheal intubation, mechanical ventilation, mortality, organophosphorus compounds, paraquat

## Abstract

Objectives

Endotracheal intubation is a critical airway intervention in severe acute poisoning, but its indications, timing, and outcomes are inconsistently described, and there are limited prospective data from Indian emergency departments (EDs). The primary objective of this prospective observational study was to determine the incidence of endotracheal intubation in adults (≥18 years) presenting with acute poisoning to the ED of AIIMS Rishikesh, Rishikesh, India. The secondary objectives were to (i) identify the clinical indications prompting intubation, (ii) enumerate indications stratified by poison type, (iii) record the timing and duration of intubation among intubated patients, and (iv) compare mortality between intubated and non-intubated patients.

Methods

A prospective observational study was conducted over 15 months (October 2024-December 2025) in the ED of AIIMS Rishikesh. Adult patients (≥18 years) presenting with acute poisoning were enrolled using consecutive sampling. Based on past departmental records, a sample size of 120 patients was estimated. Participants were followed for 28 days through hospital records or telephonic follow-up after discharge.

Results

This study included predominantly young adults with a slight male predominance (51.7%; 62/120), and the majority of poisoning cases were suicidal in nature. Pesticides, particularly organophosphorus (OP) compounds, were the most common poisoning agents, followed by household corrosives. Endotracheal intubation was required in 15.8% (19/120) of patients, with threatened airway due to a reduced Glasgow Coma Scale being the most common indication, followed by respiratory distress and excessive secretions. OP poisoning frequently required intubation due to multiple indications, while paraquat poisoning often required delayed intubation because of progressive respiratory failure. The overall mortality was 9.2% (11/120), with significantly higher mortality among intubated patients. Most deaths were attributed to OP compounds and paraquat poisoning. Male patients had higher rates of intubation and mortality.

Conclusion

This study found that 15.8% of adults with acute poisoning presenting to emergency required endotracheal intubation, with significantly higher mortality among intubated patients. OP compounds and paraquat were the leading causes of intubation and death. Most intubations were performed at presentation, emphasizing the importance of early airway assessment in the emergency while maintaining close monitoring for delayed respiratory deterioration, especially in paraquat poisoning.

## Introduction

Acute poisoning is a common reason for presentation to the emergency department (ED). Acute poisoning describes the adverse effects of a substance that result from either a single exposure or from multiple exposures in a short period of time (less than 24 hours) [1]. Poisoning can be intentional, a recreational misadventure, or accidental and can involve any number of different toxicological agents. Patients presenting with overdose may require airway support and, in some cases, intubation and mechanical ventilation [1]. The World Health Organization estimated that 640,000 people died annually due to poisoning, of which the majority of deaths occurred in low- and middle-income countries [2]. It has been estimated that, in India, five to six persons per lakh of the population die due to acute poisoning every year [3]. Poisoning is the fourth most common cause of mortality in India [4]. In 2021, global non-CO poisoning accounted for 3.58 million prevalent cases [5]. Based on the data in Mew et al. [2], approximately one in seven (110,000) of the 798,000 suicides worldwide in 2012 were the result of pesticide self-poisoning. In general, accidental poisoning is more common in children, and suicidal poisoning is more common in young adults [6].

The pattern of poisoning in a region depends on various factors, which include availability and access to the poison, socioeconomic status of an individual, cultural and religious influences, and so on [7]. In India, as agriculture is the main occupation, insecticides and other agrochemical fertilizers are used to a greater extent, and poisoning with such products is more common [8]. Endotracheal intubation remains a cornerstone of the management of the patient with severe poisoning, but little is known about the types of substances that are associated with intubation.

The intubation of a patient with an overdose for reduced consciousness, but otherwise not requiring organ support, without evidence of respiratory compromise, is controversial [9]. Considering this, we conducted a study to determine indications for intubation in acute poisoning in our setting. In previous literature, we could not find any Indian study on indications for intubation in acute poisoning. Therefore, this study aims to determine the incidence and outcomes of endotracheal intubation in patients presenting with acute poisoning in the ED of a tertiary care center.

## Materials and methods

Study design and setting

This was a single-center, prospective observational study conducted in the ED of AIIMS Rishikesh, Rishikesh, India, a tertiary care center in India.

Selection of participants

All patients presenting to the ED with acute poisoning during the study period of 15 months (October 2024-December 2025) were enrolled according to inclusion and exclusion criteria. The inclusion criteria were patients presenting to the ED with acute poisoning aged ≥18 years. The exclusion criteria were patients who were already intubated on arrival, patients who presented in cardiac arrest or required immediate resuscitation for impending cardiac arrest, and patients who were unwilling to participate in the study.

Sample size determination

A formal sample size calculation was not performed. Consecutive sampling was employed, and all eligible patients presenting to the ED with acute poisoning during the predefined 15-month study period (October 2024 to December 2025) were recruited. A total of 120 patients meeting the eligibility criteria were included in the study.

Methods and measurements

Patients presenting to the ED with acute poisoning fulfilling the inclusion criteria were enrolled in the study after providing written informed consent. The following parameters were assessed: demographic variables such as age and gender, patient details, and history. Poison-specific details included the agent of poison, group of poison, and type of exposure. Clinical parameters included vital signs on arrival, Glasgow Coma Scale (GCS), and intubation status. The GCS was assessed according to the original GCS described by Teasdale and Jennett [10]. Outcomes included duration of intubation and mortality. Incidence of endotracheal intubation was expressed in proportions and percentages. The type of exposure leading to intubation was expressed in proportions and percentages. Duration of intubation was expressed as mean ± standard deviation and median (IQR). Mortality was expressed in proportions and percentages.

Classification of poisoning agents

The poisoning agent was classified based on the available clinical history obtained from the patient or accompanying attendants, referral documentation, identification of the container or substance where available, and relevant clinical and laboratory/toxicological information. When the exact substance was not analytically confirmed, the poisoning agent was classified according to the most likely agent documented by the treating physician based on the available history and clinical presentation.

Indications and criteria for endotracheal intubation

Endotracheal intubation was performed at the discretion of the treating emergency physician based on the patient’s clinical condition. The indications for intubation were prospectively documented and categorized as threatened airway due to a low GCS of ≤8, threatened airway due to excessive secretions, respiratory distress/respiratory failure, or other clinically relevant indications. The specific indication leading to intubation was recorded at the time of the procedure. The timing of intubation was also documented as on arrival, within the predefined subsequent time intervals, as applicable.

Outcomes

The primary outcome was to determine the incidence of endotracheal intubation among adults presenting with acute poisoning. Secondary outcomes were to (i) identify the clinical indications prompting intubation, (ii) enumerate indications stratified by poison type, (iii) record the timing and duration of intubation among intubated patients, and (iv) compare mortality between intubated and non-intubated patients. Participants were followed for 28 days through hospital records or telephonic follow-up after discharge.

Statistical analysis

Data preparation was done in Microsoft Excel. Data analysis was performed using IBM SPSS Statistics for Windows, version 23.0 (IBM Corp., Armonk, NY). Descriptive statistics: proportions, percentages, means, standard deviations, and medians with IQRs. Comparative analysis: chi-square test for categorical variables and Fisher’s exact test for small sample categories. Wilcoxon-Mann-Whitney U test for non-parametric comparisons. Survival analysis: Kaplan-Meier survival curves.

Ethical approval

The study adhered to established ethical guidelines, securing ethical clearance from the Institute Ethics Committee on January 29, 2025, with approval number AIIMS/IEC/25/30. Written informed consent was obtained from all the patients before inclusion in the study. 

Compliance with manuscript writing guidelines

The manuscript was prepared in accordance with the Strengthening the Reporting of Observational Studies in Epidemiology (STROBE) guidelines [11].

## Results

A total of 120 patients who presented to the ED were included. Baseline demographics, agent of poison and circumstance are shown in Table 1.

**Table 1:** Demographic variables, agent of poison, and circumstance Data are presented as number (percentage) unless otherwise specified. Agents: acids, 26/120 (21.7%), and organophosphorus compounds, 25/120 (20.8%), together comprised roughly two-fifths of exposures, followed by aluminum phosphide, 14/120 (11.7%). Zinc phosphide accounted for 7/120 (5.8%) and paraquat for 6/120 (5.0%).

Demographics	Total, n (%)
Age (years)	
18-30 years	54 (45%)
31-40 years	36 (30%)
41-50 years	20 (16.7%)
51-60 years	7 (5.8%)
61-70 years	2 (1.7%)
71-80 years	1 (0.8%)
Gender	
Male	62 (51.7%)
Female	58 (48.3%)
Agent of poison	
Acetaminophen	3 (2.5%)
Acid	26 (21.7%
Alprazolam	1 (0.8%)
Aluminum phosphide	14 (11.7%)
Amitraz	2 (1.7%)
Antidepressants	2 (1.7%)
Benzodiazepines	5 (4.2%)
Beta-blocker	1 (0.8%)
Cannabis	6 (5.0%)
Carbon monoxide	1 (0.8%)
Chloroxylenol	5 (4.2%)
Dhatura	2 (1.7%)
Hydrochloric acid	1 (0.8%)
Lead	1 (0.8%)
Lithium	1 (0.8%)
Opioid	2 (1.7%)
Organophosphorus	25 (20.8%)
Paraquat	6 (5.0%)
Phenobarbitone	2 (1.7%)
Pyrethrin	2 (1.7%)
Super warfarin	2 (1.7%)
Tricyclic antidepressants	1 (0.8%)
Vitamins	1 (0.8%)
Yellow phosphorus	1 (0.8%)
Zinc phosphide	7 (5.8%)
Circumstance	
Accidental	2 (1.7%)
Recreational	7 (5.8%)
Suicidal	111 (92.5%)

Outcomes

Table 2 shows the primary and secondary outcomes.

**Table 2:** Primary and secondary outcomes Data are presented as number (percentage) unless otherwise specified. Continuous variables are presented as mean (SD), median (IQR), and range. GCS, Glasgow Coma Scale; SD, standard deviation; IQR, interquartile range

Outcomes	Total, n (%)
Primary outcome	
Intubation	
Yes	19 (15.8%)
No	101 (84.2%)
Secondary outcomes	
Indication of intubation	
Threatened airway (low GCS)	11 (57.9%)
Threatened airway (secretions)	3 (15.8%)
Respiratory distress	5 (26.3%)
Time of intubation	
On arrival	16 (84.2%)
From arrival to 6 hours	1 (5.3%)
From 6 to 48 hours	2 (10.5%)
Duration of intubation (days)	
Mean (SD)	1.95 (1.22)
Median (IQR)	2 (1-2)
Range	1-6
Mortality	
Yes (death)	11 (9.2%)
No (survived)	109 (90.8%)

Associations

The association of gender with intubation and mortality is shown in Table 3.

**Table 3:** Association of gender with intubation and mortality The p-values were calculated using the chi-square test.

Variable	Male (n = 62)	Female (n = 58)	Total (n = 120)	χ^2^	p-value
Intubation	16 (25.8%)	3 (5.2%)	19 (15.8%)	9.574	0.002*
Mortality	9 (14.5%)	2 (3.4%)	11 (9.2%)	4.409	0.036*

There was a significant difference between the two genders in terms of the distribution of intubation (χ² = 9.574, p = 0.002). Male participants had a higher proportion of intubation compared to female participants, and this was significantly associated (p = 0.002).

There was a significant difference between the two genders in terms of the distribution of mortality (χ² = 4.409, p = 0.036). Among the male participants, 14.5% (9/62) had a fatal outcome. Among the female participants, 3.4% (2/58) had a fatal outcome. Male participants had a larger proportion of mortality compared to female participants, and this was significantly associated (p = 0.036).

Association between indication and timing of intubation is depicted in Figure 1.

**Figure 1:**
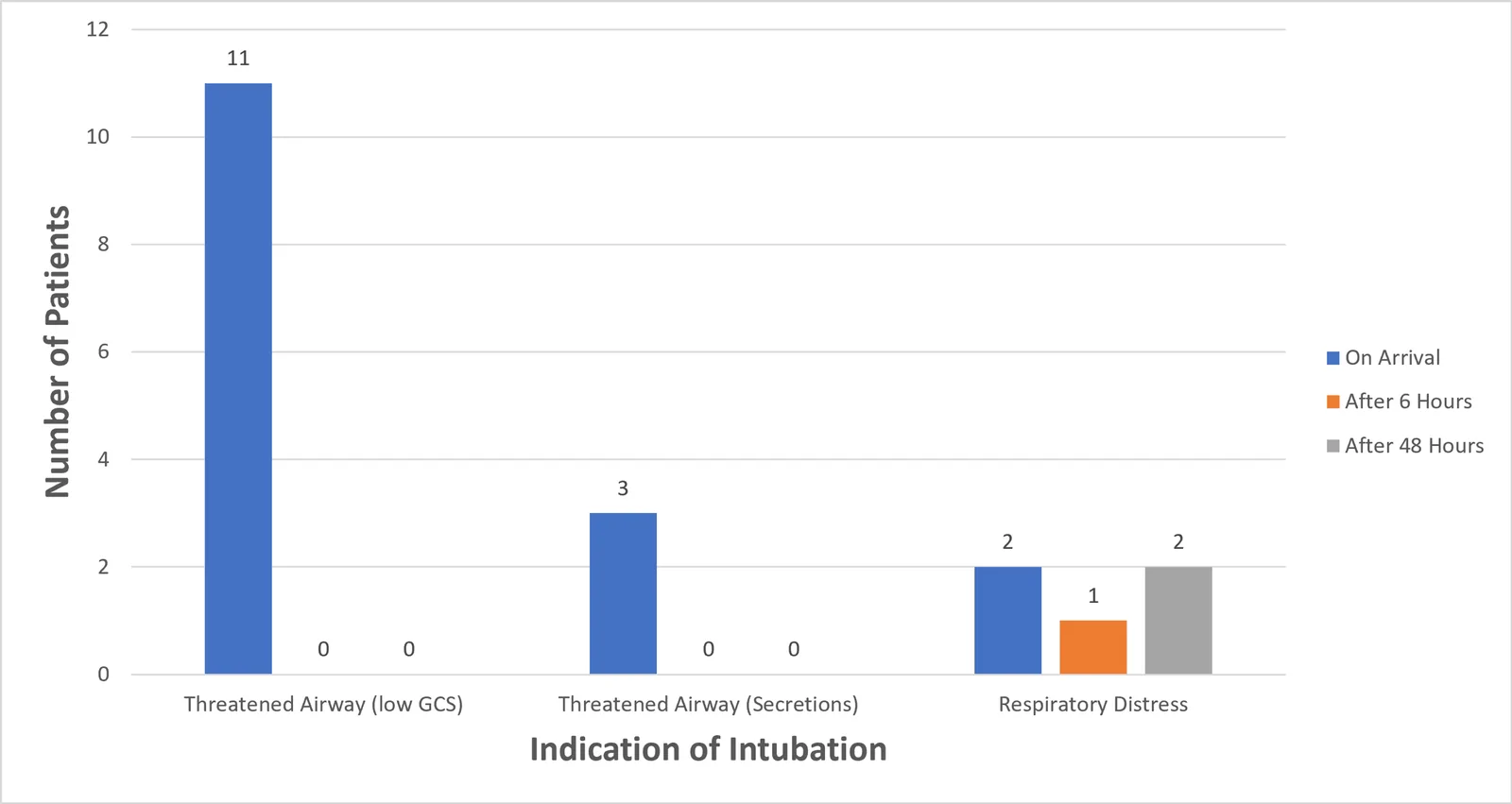
Distribution of timing of endotracheal intubation according to indication for intubation Bars show the number of patients intubated per indication, stratified by timing (on arrival, after 6 hours, and after 48 hours); values are expressed as number. GCS = Glasgow Coma Scale

Threatened airway due to low GCS (11/11, 100%) and threatened airway due to excessive secretions (3/3, 100%) were exclusively managed with endotracheal intubation on arrival to the ED. In contrast, among patients intubated for respiratory distress (n = 5), two (40%) were intubated on arrival, one (20%) was intubated after six hours, and two (40%) were intubated after 48 hours of presentation.

Incidence of intubation and mortality across different agents of poison is depicted in Figure 2 and Table 4.

**Figure 2:**
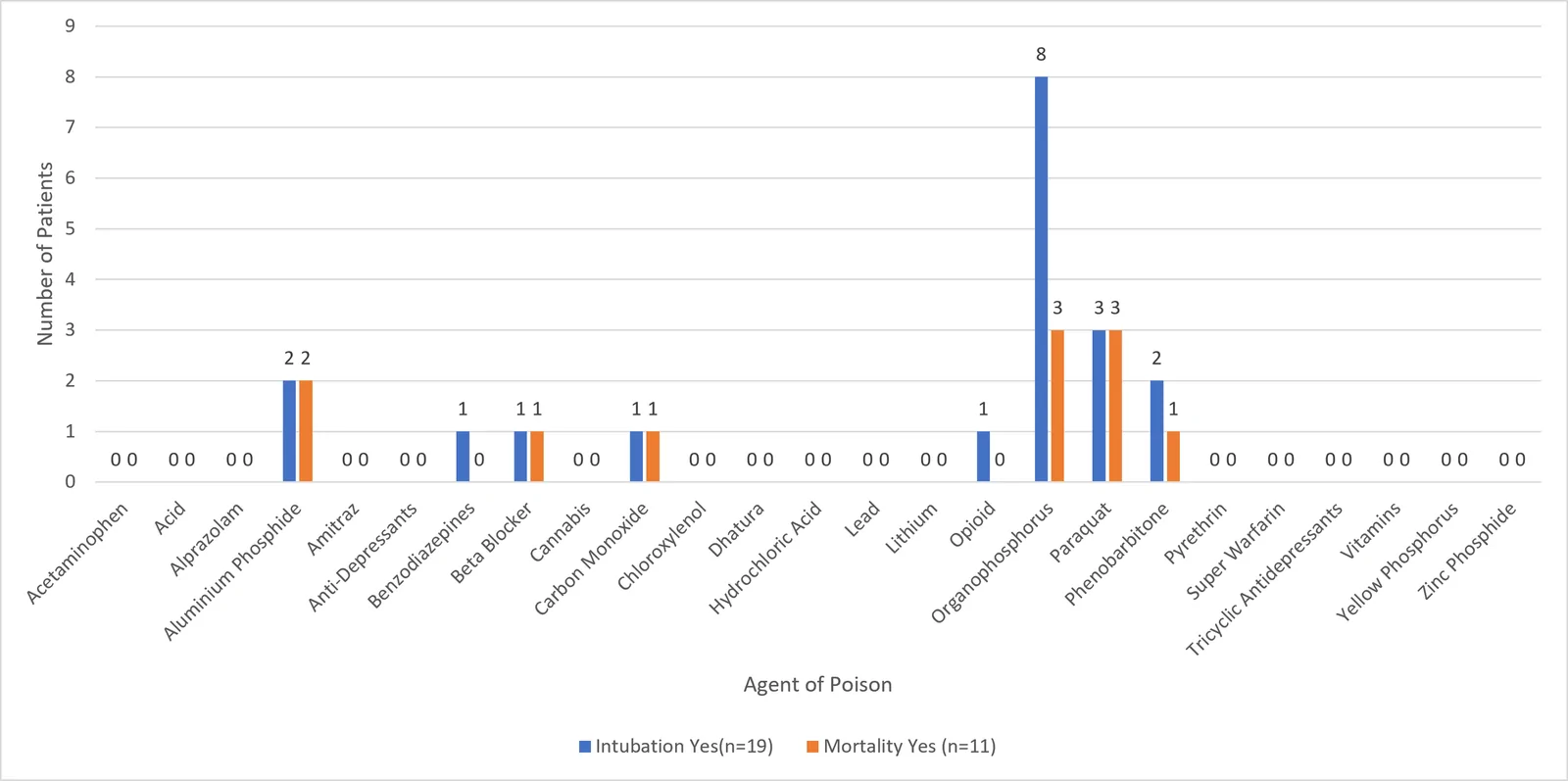
Incidence of intubation and mortality across different agents of poison Data are presented as number unless otherwise specified.

**Table 4:** Distribution of poisoning agents and their association with intubation and mortality Fisher’s exact test demonstrated a statistically significant association between poisoning agent and need for endotracheal intubation (p = 0.005) and between poisoning agent and mortality (p = 0.042).

Agent of poison	Total, n (%)	Intubation, n (%)	Mortality, n (%)
Acetaminophen	3 (2.5)	0 (0.0)	0 (0.0)
Acid	26 (21.7)	0 (0.0)	0 (0.0)
Alprazolam	1 (0.8)	0 (0.0)	0 (0.0)
Aluminum phosphide	14 (11.7)	2 (10.5)	2 (18.2)
Amitraz	2 (1.7)	0 (0.0)	0 (0.0)
Antidepressants	2 (1.7)	0 (0.0)	0 (0.0)
Benzodiazepines	5 (4.2)	1 (5.3)	0 (0.0)
Beta-blocker	1 (0.8)	1 (5.3)	1 (9.1)
Cannabis	6 (5.0)	0 (0.0)	0 (0.0)
Carbon monoxide	1 (0.8)	1 (5.3)	1 (9.1)
Chloroxylenol	5 (4.2)	0 (0.0)	0 (0.0)
Dhatura	2 (1.7)	0 (0.0)	0 (0.0)
Hydrochloric acid	1 (0.8)	0 (0.0)	0 (0.0)
Lead	1 (0.8)	0 (0.0)	0 (0.0)
Lithium	1 (0.8)	0 (0.0)	0 (0.0)
Opioid	2 (1.7)	1 (5.3)	0 (0.0)
Organophosphorus	25 (20.8)	8 (42.1)	3 (27.3)
Paraquat	6 (5.0)	3 (15.8)	3 (27.3)
Phenobarbitone	2 (1.7)	2 (10.5)	1 (9.1)
Pyrethrin	2 (1.7)	0 (0.0)	0 (0.0)
Super warfarin	2 (1.7)	0 (0.0)	0 (0.0)
Tricyclic antidepressants	1 (0.8)	0 (0.0)	0 (0.0)
Vitamins	1 (0.8)	0 (0.0)	0 (0.0)
Yellow phosphorus	1 (0.8)	0 (0.0)	0 (0.0)
Zinc phosphide	7 (5.8)	0 (0.0)	0 (0.0)
Total	120 (100.0)	19 (100.0)	11 (100.0)

There was a significant difference between the intubated and not intubated groups in terms of distribution of agent of poison (p = 0.005). Three agents dominated: organophosphorus (OP), paraquat, and aluminum phosphide accounted for 68.4% of all intubations. OP was the leading agent responsible for 42% (8/19) of all intubations, with 32% (8/32) of cases requiring intubation out of all OP cases. Paraquat posed the highest per-case risk: 50% (3/6) intubation rate; every second patient required intubation. Despite acid being the most common agent (26 cases), no patients with acid poisoning required intubation.

There was a significant difference between the death and survival groups in the distribution of agent of poison (p = 0.042). The top three agents causing mortality were paraquat (27.3%; 3/11), OP (27.3%; 3/11), and aluminum phosphide (18.2%; 2/11). Paraquat was found to be the deadliest, with 50% mortality; paraquat posed the highest per-case death risk.

Survival analysis

Figure 3 shows the survival analysis for intubation by Kaplan-Meier curve.

**Figure 3:**
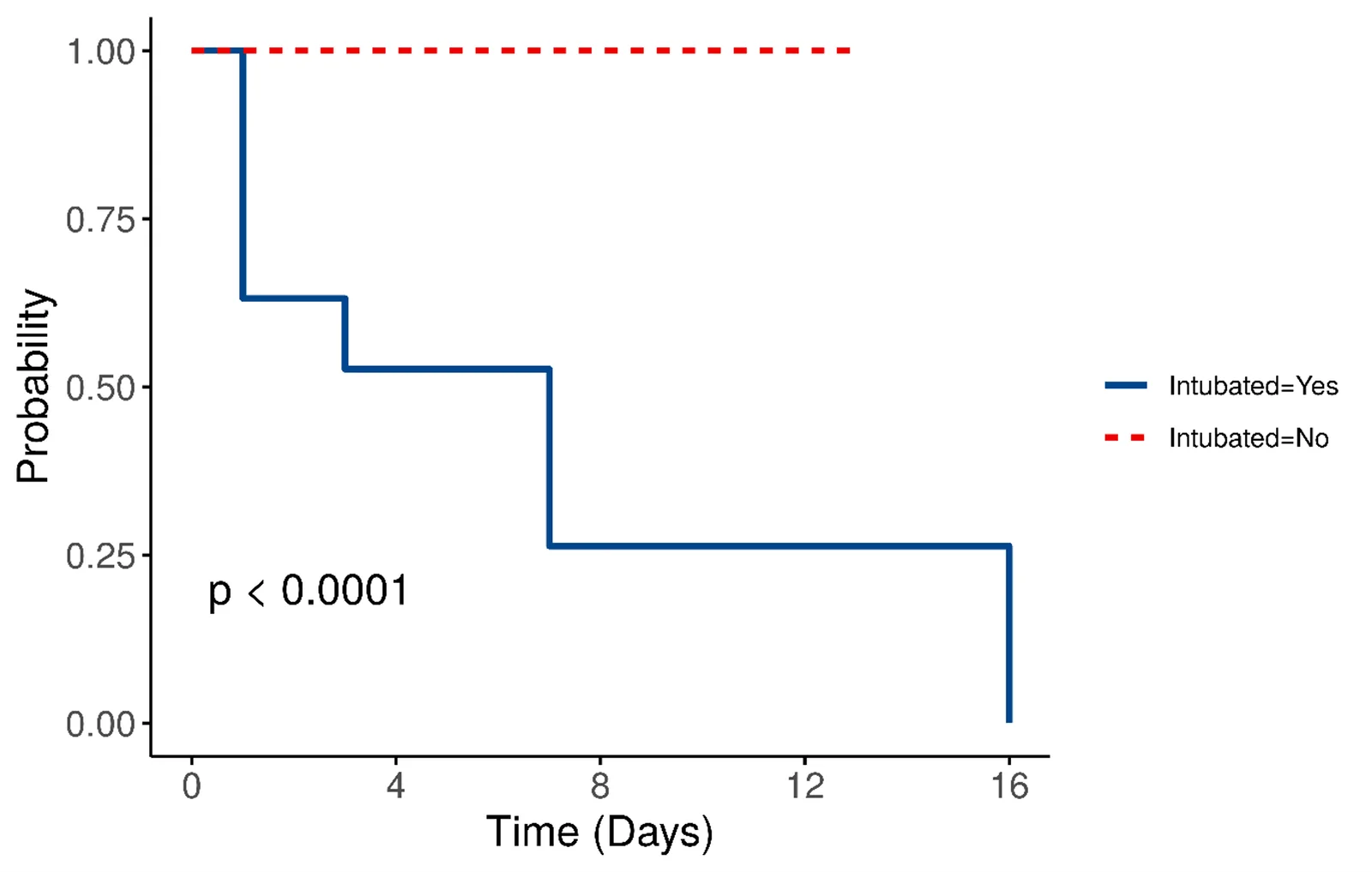
Kaplan-Meier curve: survival analysis for intubation The p-values were calculated using the log-rank test. Intubated (solid blue line): this group shows a rapid decrease in survival probability, dropping to 0.25 by day 8; it continues to drop until day 16. Non-intubated (dashed red line): this group maintains a survival probability of 1.00 throughout. The data suggest that patients who were not intubated had a statistically significantly higher survival probability over the 16 days compared to those who were intubated.

## Discussion

This study describes a single-center cohort of adults with acute poisoning presenting to the ED over the study period. The age distribution was skewed toward young adults with a mean age of 33.68 ± 11.34 years and a median of 32 years (IQR: 24.75-40.25; range: 18-72). It was similar to a previous study by Srivastava et al. (2005) conducted at AIIMS Delhi, New Delhi, India, which showed the highest incidence in the range of 14-40 years. In a study by Sharma et al. (2019) in Northeast India, the mean age was 28.43 ± 14 years, with the highest incidence in the range of 21-30 years [12,13]. The study population predominantly comprised young adults, with a median age of 32 years, indicating that acute poisoning in the present setting primarily affected individuals in the younger age group. In terms of gender distribution, we encountered a slight male preponderance (51.7% male vs. 48.3% female), which was not statistically significant, indicating a relatively balanced gender distribution. Previous studies done by Srivastava et al., Sharma et al., and Beauchamp et al. showed significant male predominance [12-14].

In terms of agents of poison, acids (26/120; 21.7%) and OP compounds (25/120; 20.8%) together comprised roughly two-fifths of exposures, followed by aluminum phosphide (14/120; 11.7%). Zinc phosphide accounted for 7/120 (5.8%) and paraquat for 6/120 (5.0%). OP compounds were the most frequently encountered poisoning agents, followed by aluminum phosphide and paraquat, reflecting the poisoning profile observed in the study setting. The predominance of pesticide poisoning may be related to their availability and accessibility within the surrounding community. Previous studies conducted by Kumar et al. in Andhra Pradesh, India, and a study conducted by Jawarkar et al. in the Amaravati district of Maharashtra, India, showed OP as the most common agent [15,16]. When grouped by broader toxicological classes, pesticides were the largest common group (49.1%; 59/120), with insecticides forming the largest category (23.3%; 28/120), followed by rodenticides (20.8%; 25/120), and herbicides (5.0%; 6/120), followed by household toxins (21.7%; 26/120). In contrast, previous studies like Srivastava et al. conducted at AIIMS Delhi showed the highest incidence due to household toxins, whereas pesticides were found to be common in a study conducted by Sharma et al. in India and in studies conducted in Sri Lanka and Thailand by Wananukul et al. and Fernando, respectively [12,13,17,18].

Endotracheal intubation was undertaken in 19 patients, giving an intubation incidence of 15.8% (19/120) in the studied cohort, which was consistent with Burket et al. (2021), in which approximately 16% of all adult overdoses were intubated [19]. Studies on OP poisoning conducted by Unnikrishnan and Shenoy (2012) in Udupi district, India, showed an intubation rate of 49.15%, and Sungur and Güven (2001) in Turkey showed an intubation rate of 21.2% [4,20]. In our study, 32% (8/25) of patients with OP poisoning required intubation. Though indications for intubation were similar to those encountered in various emergency patients, indications for various agents reflected their classic toxidromes. Threatened airway due to low GCS was the leading driver (57.9%; 11/19), followed by respiratory distress (26.3%; 5/19) and threatened airway from copious secretions (15.8%; 3/19). Timing was predominantly immediate: 84.2% (16/19) were intubated on arrival in the ED, while delayed intubations occurred from arrival to six hours (5.3%; 1/19) or from six to 48 hours (10.5%; 2/19). No prior studies evaluating the timing of intubation were identified. The duration of invasive ventilation was relatively brief, with a mean of 1.95 ± 1.22 days and a median of two days (IQR: 1-2; range: 1-6). In a study on OP poisoning by Sungur and Güven, the duration of mechanical ventilation was 4.1 ± 3.2 days [20].

In our study cohort, mortality was 9.2% (11/120), while previous studies done by Sharma et al. in Northeast India on all poisoning agents showed an 8.3% mortality. The mortality rate was 5.5% in a study conducted in Thailand by Fernando on all poisoning agents, while a study conducted by Kaeley et al. at our own center on acute poisoning in the geriatric population showed a mortality rate of 16.9% [13,18,21].

Common agents of poisoning encountered in females were acids, household toxins, and rodenticides (found in households), whereas males had higher proportions of herbicides such as paraquat and insecticides. The majority of poisoning cases occurred under suicidal circumstances, accounting for 92.5% (111/120) of all cases, which was similar to previous studies: Getie and Belayneh (2020) conducted in Northeast Ethiopia showed 64.2%, Sulaj et al. (2015) conducted in Albania showed 87.2%, and Sharma et al. conducted in Northeast India showed 63.96% [13,22,23]. Our study showed a higher suicidal percentage. Males were significantly more likely to be intubated (25.8% vs. 5.2% in females). This may reflect more severe poisoning presentations in males and ingestion of agents with greater respiratory or neurological toxicity. The higher intubation rate in males is consistent with the higher prevalence of poisoning using high-toxicity agents such as OP compounds and paraquat.

Male mortality (14.5%) significantly exceeded female mortality (3.4%). This is likely driven by higher rates of high-fatality agents (herbicides: paraquat and OP compounds) among males, which increased the need for intubation and complications. In all, 57.9% (11/19) of intubated patients died; no deaths occurred in the non-intubated group in our study conducted on all poisoning agents. In OP poisoning, 12% (3/25) mortality was observed. In previous literature on OP poisoning, Sungur and Güven showed 50% mortality in intubated patients, while Unnikrishnan and Shenoy showed 38% mortality in intubated patients [4,20]. Indication for intubation: OP poisoning has multiple indications, such as low GCS, secretions, and respiratory distress, showing its severity by involving multiple systems. All patients with paraquat poisoning who were intubated were due to respiratory distress. The respiratory distress did not occur at arrival but developed within six hours, requiring intubation, and some required intubation between six and 48 hours, showing gradual worsening lung injury. Paraquat and OP compounds accounted for the majority of deaths. The higher mortality observed among patients requiring endotracheal intubation should be interpreted cautiously. Patients who require intubation are likely to have more severe poisoning and greater physiological derangement, which may itself account for the increased mortality. Therefore, in this observational study, intubation should be regarded primarily as a marker of illness severity rather than interpreted as a direct cause of mortality. The observed association does not establish a causal relationship between intubation and mortality.

Limitations

The authors acknowledge the following limitations in the study. First, the study is a single-center design. It was conducted only at AIIMS Rishikesh, a tertiary care center in India. It may not represent poisoning patterns in other regions (urban vs. rural, different states). Secondly, a smaller sample size (n = 120) limits statistical power. Many poisoning agents had only one to two cases, so we may miss clinically important differences. Thirdly, OP poisoning numbers may reflect the agricultural context of the study setting, and that may limit generalizability to urban populations, where patterns of toxic exposure may differ. Fourthly, the study included only adults (18+), so it cannot be applied to pediatric emergency care.

## Conclusions

This prospective observational study of 120 consecutive adult poisoning cases at AIIMS Rishikesh establishes critical benchmarks for airway management and outcomes in Indian emergency toxicology. Our 15.8% (19/120) intubation rate aligns with international data, validating the global consistency of severe poisoning thresholds across diverse healthcare contexts. The mortality differential, 57.9% (11/19) among intubated patients versus zero deaths in non-intubated cases, reveals that intubation serves not merely as therapy but as a powerful prognostic marker identifying patients at imminent risk of death. This binary outcome pattern represents a critical inflection point in poisoning management. Our data demonstrate clear toxicological risk stratification. OP compounds and paraquat drove both respiratory failure and mortality despite representing only one-quarter of exposures, while household acids, rodenticides, and most pharmaceuticals caused no deaths despite numerical predominance. This concentration of severe outcomes argues for agent-specific protocols rather than uniform approaches.

Endotracheal intubation was required in a proportion of patients presenting with acute poisoning, with airway protection and respiratory compromise being important indications. Patients requiring intubation had higher mortality; however, this association should be interpreted in the context of the greater severity of poisoning among these patients and does not establish a causal relationship between intubation and mortality. The temporal pattern of 84% (16/19) of intubations occurring immediately on arrival compresses the assessment window to initial patient contact, establishing rapid airway evaluation and emergency intubation as core frontline competencies rather than specialist procedures. Emergency physicians should adopt an early, poison-specific approach to airway assessment that integrates neurological status, respiratory function, and toxin characteristics. Close monitoring for delayed respiratory deterioration, particularly in paraquat poisoning, and timely escalation of care may improve clinical outcomes.
